# Where are my instruments? Hazards in delivery of surgical instruments

**DOI:** 10.1007/s00464-015-4537-7

**Published:** 2015-10-20

**Authors:** Annetje C. P. Guédon, Linda S. G. L. Wauben, Anne C. van der Eijk, Alex S. N. Vernooij, Frédérique C. Meeuwsen, Maarten van der Elst, Vivian Hoeijmans, Jenny Dankelman, John J. van den Dobbelsteen

**Affiliations:** Department of BioMechanical Engineering, Faculty of Mechanical, Maritime and Materials Engineering, Delft University of Technology, Mekelweg 2, 2628 CD Delft, The Netherlands; Leiden University Medical Center, Albinusdreef 2, 2333 ZA Leiden, The Netherlands; Reinier de Graaf Groep, Reinier de Graafweg 3-11, 2625 AD Delft, The Netherlands

**Keywords:** Surgical instruments, Safety, Risk analysis, Information technology, Logistics

## Abstract

**Background:**

Unavailability of instruments is recognised to cause delays and stress in the operating room, which can lead to additional risks for the patients. The aim was to provide an overview of the hazards in the entire delivery process of surgical instruments and to provide insight into how Information Technology (IT) could support this process in terms of information availability and exchange.

**Methods:**

The process of delivery was described according to the Healthcare Failure Mode and Effects Analysis methodology for two hospitals. The different means of information exchange and availability were listed. Then, hazards were identified and further analysed for each step of the process.

**Results:**

For the first hospital, 172 hazards were identified, and 23 of hazards were classified as high risk. Only one hazard was considered as ‘controlled’ (when actions were taken to remove the hazard later in the process). Twenty-two hazards were ‘tolerated’ (when no actions were taken, and it was therefore accepted that adverse events may occur). For the second hospital, 158 hazards were identified, and 49 of hazards were classified as high risk. Eight hazards were ‘controlled’ and 41 were ‘tolerated’. The means for information exchange and information systems were numerous for both cases, while there was not one system that provided an overview of all relevant information.

**Conclusions:**

The majority of the high-risk hazards are expected to be controlled by the use of IT support. Centralised information and information availability for different parties reduce risks related to unavailability of instruments in the operating room.

The operating room (OR) is known to be the most cost-intensive place of the hospital where adverse events are most likely to occur [1–9]. Weaknesses in the hospital organisation; lack of experience of the OR team; limitations in checklists, protocols, and in equipment design allow adverse events to occur in a complex environment such as the OR [10]. Because of the increasing use of technology during surgery and the added complexity it induces, an increase in equipment-related incidents has been reported [6, 11–13]. Equipment-related incidents were observed in 15.9 % of surgical procedures [6]. Around 40 % of these incidents were due to the unavailability of equipment [6, 11], mostly instruments, and each incident resulted in an average of 12 min of extra work and 5 min of delay [6]. Verdaasdonk et al. have observed a larger number of incidents specifically related to instruments in 16 % of the procedures [12]. Equipment-related issues have also shown to increase the level of stress of the surgeon [14]. Stress is known to diminish human performances and therefore increase the potential for errors in the OR [14, 15]. Hence, managing stress-inducing factors is imperative for safer care. Moreover, a higher percentage of incidents was observed during orthopaedic procedures [6], which is considered particularly disruptive, because these surgeries highly depend on the availability and function of procedure-specific instruments [13]. These studies show that equipment-related problems represent a large part of the adverse events in the OR, and that optimisation of the supply chain is likely to have a large impact on patient safety. However, although unavailability of instruments is recognised to cause delays and stress in the OR, the processes related to the delivery of instruments have received little attention in the scientific literature.

Supply chain management is also a topic of increased interest due to the increased emphasis on cost efficiency in health care. Hospitals are fused and may share centralised services, while others outsource services to focus on their primary processes: patient cure and care. Outsourcing the sterilisation of surgical instruments presents opportunities to reduce costs if the processes are well designed and optimised. However, poor supply chain management can lead to unavailability of instruments and therefore present risks for patients [16]. Information exchange and trust have been identified as important factors that influence the quality of supply chain management [17]. Centralising information is in this case imperative as it increases the availability and ease of access for different parties, thereby enhancing the collaboration between them [18]. Both information exchange and trust can be supported with currently available applications in Information Technology (IT). In particular, recent developments such as ‘track and trace’ methods have the potential to extensively improve inventory management and streamline processes in health care [19]. Moreover, stricter requirements are being set for the ability to trace the use of medical equipment in case of a recall due to malfunctions or contamination [20]. Still, the potential added value of IT support for improvement and optimisation of supply chain management is unclear.

The organisational structure (e.g. outsourced or shared centralised services), information exchange, and trust between the different parties all influence the delivery process of instruments [16, 17]. Each of these can induce hazards (i.e. sources of potential adverse event) at different stages of the delivery process, although this may only become apparent during the procedure. As far as known by the authors, an overview of hazards in the entire process of delivery of instruments is lacking in the scientific literature. One way to obtain such an overview is through the application of a Healthcare Failure Mode and Effects Analysis (HFMEA). The first HFMEAs were performed in the mid-1960s in the aviation industry and are nowadays used by many high-risk industries. HFMEAs allow identification of (previously unnoticed) errors and supports meeting high safety standards. Several adaptations of this method have been developed and successfully applied in the health care sector [21–23]. This study aims to provide an overview of the hazards in the entire process of delivery of surgical instruments using the HFMEA method and to provide insight into how IT could support this process in terms of information availability and exchange. As an exemplary case, we focus on planned orthopaedic surgeries, because of the large amount of instrument trays needed during orthopaedic procedures and the frequent use of loaned instrument trays (i.e. specific sets of instruments provided by vendors when needed). We analysed the delivery process of loaned instrument trays in particular, as it is a more extensive process compared to instrument trays owned by the hospital.

## Materials and methods

### Hospital settings and participants

The Healthcare Failure Mode and Effects Analysis (HFMEA) was performed in two Dutch hospitals with different organisational structures. The first case is an academic hospital with an internal Central Sterilisation Service Department (CSSD) located in the hospital one floor below the OR complex. The second is a teaching hospital with an outsourced CSSD located a few kilometres from the hospital.

The HFMEA performed in this study follows the guidance of a safety programme for Dutch hospitals [24]. It was completed in six sessions of approximately two hours. In between sessions, email communication was used to share relevant documentation, and some individual meetings were planned for discussions on specific steps of the process. Before the first session, the focus of the HFMEA was defined in both hospitals, which was the entire process of delivery of loaned trays for orthopaedic surgeries, from the moment the surgeon decides that loaned instruments are needed for a patient until the return shipment to the vendors.

A multidisciplinary team of eight team members and two HFMEA facilitators was formed for both hospitals. The team members were chosen such that all parties relevant for the process of delivery of loaned trays from the hospitals’ point of view were represented. In the first case, the team consisted of one orthopaedic surgeon, two OR nurses, one OR administrator, one OR quality advisor, one CSSD employees, one OR equipment specialist, and one manager of the purchase department. For the second case, the team consisted of one orthopaedic surgeon, two OR nurses, one OR team leader, one CSSD employee, one CSSD manager, and one scheduler for orthopaedic surgeries.

### Process

In the first two sessions, the process of delivery of loaned trays was described by the HFMEA team and translated in a flow diagram (including main steps and sub-steps). The teams also provided an estimation of the time needed for each sub-step of the process. After the sessions, the different means of information exchange and availability during the processes were listed for the two cases.

### Hazard analysis

In the third and fourth sessions, potential ‘failure modes’ and their causes were identified for each (sub)step of the process. Each combination of a failure mode and cause was considered to be a hazard. In the last two sessions, a score was attributed to each hazard for their occurrence (O) and severity (S) according to the hazard scoring matrix in Table 1. The rating and meaning of the scores were based on the Dutch guide for risk analysis [24], but were slightly adapted by the HFMEA teams to describe the occurrence and severity related to the delivery of instrument trays. Both scores were multiplied and provided the risk score (*R* = *O* × *S*). A list of high-risk hazards was provided by selecting the hazards with a risk score equal or higher than 10 or a severity equal or higher than 4. Finally, the team determined whether the hazards were ‘tolerated’ or ‘controlled’ and provided recommendations for future improvements. A hazard was considered as ‘tolerated’ when the HFMEA team agreed that no actions were taken later in the process to remove the hazard, and it was just accepted that the adverse event may occur. On the contrary, a hazard was considered as ‘controlled’ when it becomes visible and can be eliminated at a later stage in the process. For instance, a hazard can be controlled by providing the needed information when needed or by actions such as an automatic control or a double check.

**Table 1:** Hazard scoring matrix

Rating	Occurrence (O)	Severity (S)
1	Never	No influence
2	Rare (less than once every 3 months)	Alternative routine, no consequences for patient
3	Occasional (more than once every 3 months)	Alternative routine, minor consequences for patient
4	Frequent (more than once a month)	Surgery is delayed/cancelled, temporary consequences for patient
5	Often (more than once a week)	Surgery is delayed/cancelled, serious consequences for patient

After the six sessions, the authors selected the high-risk hazards that could be controlled by the use of IT support to centralise, store, and exchange information. In this study, IT support includes the following features:

- A digital OR schedule taking the availability of instrument trays into account
- Information on the necessity of (loaned) instrument trays for each surgery
- Information on the status of (loaned) instrument trays (order, delivery, sterilisation, transport, use in OR, etc.). This information could partly be provided by a ‘track and trace’ system for instrument trays. Tracking and tracing of single instruments is not taken into account in this study, as the technology is not yet fully implemented in hospitals.

## Results

### Process

#### Case I: Hospital with internal CSSD

**Fig. 1:**
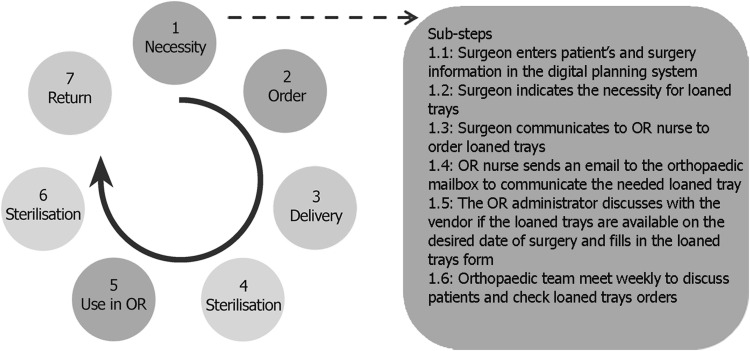
Entire process of delivery of loaned trays in the case of internal CSSD (*left*) and sub-steps of the first step ‘Necessity’ (*right*). Steps 1, 2, and 5 (*in orange*) are performed mainly by the OR staff; steps 3 and 7 (*in blue*) are performed by the vendor; and steps 4 and 6 (*in green*) by the CSSD

The entire process for the hospital with internal CSSD consisted of seven main steps and 57 sub-steps and had a duration of 690 min (see Table 4). The main steps and an example of sub-steps are shown in Fig. 1.

- Step 1: The orthopaedic surgeon determines the necessity for loaned instrument trays and communicates this to an OR nurse who write down the information in an email. The OR administrator contacts the supplier to reserve the loaned trays for the specific date and fills out an ordering form. The procedures for which loaned instrument trays are needed are discussed each week with a surgeon and an OR nurse.
- Step 2: First, the order is placed in the ordering system of the hospital and then at the vendor. An overview of the orders is sent to the CSSD and is printed.
- Step 3: The vendor delivers the instrument trays at the CSSD, and the content is then checked by the CSSD employee.
- Step 4: The instrument trays are cleaned and sterilised at the CSSD.
- Step 5: The instrument trays are transported to the OR complex, set out according to the OR schedule, and finally used during the surgical procedure. After surgery, the instruments are counted and placed back in the trays to be sent back to the CSSD.
- Step 6: The instrument trays are transported back to the CSSD, are cleaned, and are sterilised.
- Step 7: The supplier collects the instrument trays at the CSSD. The OR administrator updates the used materials in the ordering system of the hospital, and the supplier sends an invoice to the hospital.

The different means of information exchange during the entire process are shown in Table 2, and the different systems where information was available are shown in Table 3.

**Table 2:** Different means of information exchange during the entire process

	Case I	Case II
Oral communication between two persons	6	4
Action to transfer information into digital systems (digital forms, emails, barcodes)	18	13
Action to transfer information into written systems (written forms, written agenda, planning overview on whiteboard, prints, faxes, labels)	10	12

**Table 3:** Different systems where information was available during the process

	Case I	Case II
The digital patient planning system of the hospital	✓	✓
Mailboxes	✓ (3)	✓ (2)
A written form to order loaned trays	✓	
A digital form to order loaned trays		✓
A planning overview on a whiteboard in OR complex for the orthopaedic team	✓	
A paper agenda for the orthopaedic team		✓
The digital ordering system of the hospital	✓	✓
A map with printed orders of the OR	✓	✓
A paper agenda and whiteboard of the CSSD	✓	✓
A map with printed orders of the CSSD		✓
Barcodes and labels on instrument trays	✓	✓
The digital system of CSSD	✓	✓
A delivery overview on a whiteboard in the OR complex		✓
A fax from the CSSD to the OR		✓
A form for used implants during surgery	✓	✓
A certification of decontamination	✓	✓

#### Case II: Hospital with external CSSD

**Fig. 2:**
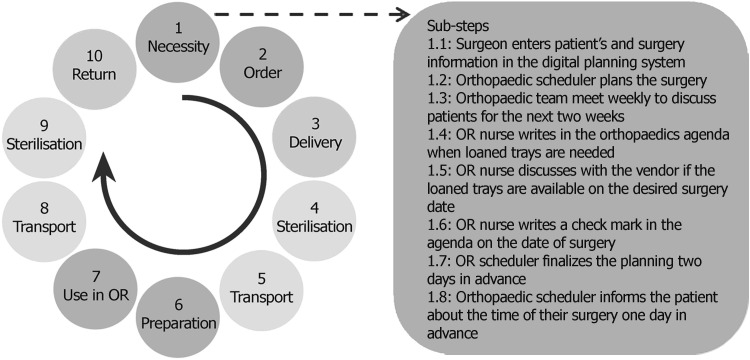
Entire process of delivery of loaned trays in the case of external CSSD (*left*) and sub-steps of the first step ‘Necessity’ (*right*). Steps 1, 2, 6, and 7 (*in orange*) are performed mainly by the OR staff; steps 3 and 10 (*in blue*) are performed by the vendor; and steps 4, 5, 8, and 9 (*in green*) by the CSSD

The entire process for the hospital with external CSSD consisted of 10 main steps and 71 sub-steps and had a duration of 715 min (see Table 4). The main steps and an example of sub-steps are shown in Fig. 2.

**Table 4:** Results of hazard analysis for both cases

Main step	Sub-steps		Time (min)		Hazards		High-risk hazards		High-risk hazards currently controlled		High-risk hazards that could be controlled by IT support	
Case	I	II	I	II	I	II	I	II	I	II	I	II
Necessity	6	8	125	130	29	37	11	16	0	2	9	14
Order	7	8	50	45	20	14	1	4	0	0	1	3
Delivery	8	8	110	70	27	28	4	4	0	3	2	1
Sterilisation	6	6	100	95	5	8	1	2	0	1	1	2
Transport	–	4	–	60	–	4	–	1	–	0	–	0
Preparation	–	8	–	95	–	20	–	7	–	1	–	6
Use in OR	11	9	100	55	49	26	4	13	1	0	2	4
Transport	–	4	–	55	–	1	–	0	–	0	–	0
Sterilisation	10	8	135	95	22	9	1	2	0	1	0	1
Return	9	8	70	15	20	11	1	0	0	0	1	0
Total	57	71	690	715	172	158	23	49	1	8	16 (70 %)	31 (63 %)
Total risk score					813	1096	258	510

- Step 1: The orthopaedic surgeon determines the necessity for loaned trays. The surgical procedures requiring loaned trays are discussed each week with a surgeon, an OR nurse, the OR team leader, and the orthopaedic scheduler. An OR nurse notes in an agenda when the loaned trays are requested and contacts the supplier to reserve the loaned trays for the specific date.
- Step 2: An OR nurse fills in an ordering form and transmits the information to the OR purchaser who places the order in the ordering system of the hospital. The CSSD is informed about the order and updates the information in their own paper files.
- Step 3: The vendor delivers the instrument trays at the CSSD, and the content is then checked by the CSSD employee.
- Step 4: The instrument trays are cleaned and sterilised at the CSSD.
- Step 5: The instrument trays are delivered to the hospital.
- Step 6: The delivery is checked, and the instrument trays are set out according to the OR schedule.
- Step 7: The instrument trays are used during the surgical procedure. After surgery, the instruments are counted and placed back in the trays to be sent back to the CSSD.
- Step 8: The instrument trays are transported back to the CSSD.
- Step 9: The instrument trays are cleaned and sterilised at the CSSD.
- Step 10: The supplier collects the instrument trays at the CSSD, checks the content, and sends an invoice to the hospital, which is later checked by the OR purchaser.

Again, the different means of information exchange during the entire process are shown in Table 2, and the different systems where information was available are shown in Table 3.

### Hazard analysis

The hazards found for both hospitals were very diverse. Examples of high-risk hazards when the surgeon is entering patient’s and surgery information in the digital planning system (step 1.1 in both cases) are: ‘Information about the surgery (type of implant and needed equipment) is not complete in the digital patient planning system’ and ‘Information is not correctly filled out in the digital patient planning system, because of lack of knowledge of the surgeon in training’. Examples of high-risk hazards during the weekly meeting of the orthopaedic team (step 1.6 for case I and step 1.3 for case II) are: ‘No overview of the defect instrument trays’ and ‘Patient is scheduled and operated in between two meetings’.

The hazards that could be controlled by the use of IT support are diverse as well. An example of such a high-risk hazard is: ‘A tray is double booked for a surgery, because within one discipline (e.g. orthopaedic surgery), there is no knowledge about the planning of another discipline (e.g. trauma surgery)’. A digital OR schedule, taking the availability of instrument trays into account, could control this hazard. Another example is: ‘A tray is not available just before the start of the surgery, because it was used for an emergency surgery’. This hazard could be controlled by tracking and tracing information that would identify directly when an instrument tray is taken to another OR and would automatically inform the responsible person.

#### Case I: Hospital with internal CSSD

One hundred seventy-two hazards were identified, and 23 of them were defined as high risk (Table 4). Only one hazard was considered as ‘controlled’ later in the process, the other 22 hazards were considered as ‘tolerated’. Sixteen high-risk hazards could be controlled by the use of IT support.

#### Case II: Hospital with external CSSD

One hundred fifty-eight hazards were identified, and 49 of them were defined as high risk (Table 4). Eight hazards were considered as ‘controlled’ later in the process, the other 41 hazards were considered as ‘tolerated’. Thirty-one high-risk hazards could be controlled by the use of IT support.

## Discussion

Using the HFMEA methodology, an overview of the hazards involved in the entire process of delivery of loaned instruments for orthopaedic surgery was provided for two hospitals with different organisational structures (internal and external CSSD). The results showed a higher number of main process steps for the hospital with external CSSD caused by the transportation to and from the OR. Furthermore, the process step ‘Preparation’ was added for case II just before the ‘Use in the OR’. The HFMEA team made this decision, because the number of sub-steps would have been too high to describe in one process step. However, the time needed for the entire process was comparable. The hazards analysis showed that the first case presented the most hazards, but less high-risk hazards. When focussing on the high-risk hazards, the largest differences in numbers between the two cases were found for the process steps ‘Necessity’, ‘Preparation’, and ‘Use in OR’. The same holds for the differences in numbers of hazards that could be controlled by IT support. The larger number of high-risk hazards observed for case II for these process steps brings more opportunities to control these hazards. Van de Klundert et al. pointed out that outsourcing of the CSSD may induce a higher risk of instruments unavailability and increased costs depending on the extend of logistics optimisation [16]. This is in line with the highest total risk scores found for case II, which could probably be improved by optimising the supply chain.

Although the observed differences between the two cases are remarkable, the results do not allow us to draw conclusions on what approach is safer. HFMEA is considered to be a strong tool for qualitative analysis [25]. It provides insight into the different types of hazards and how the risks could be minimised. However, despite the structured approach of the HFMEA and the scoring of the hazards by each individual team member prior to the sessions, the determination and scoring of hazards still depends on the opinion of the team members and is as such susceptible to subjectivity [26]. Nevertheless, this method enhances awareness among the team members as well as communication and cooperation between the different hospital areas [25, 26]. As such, this study provides insight into the type of hazards observed in both hospitals and how IT could support the delivery of surgical instruments.

Regarding the means of information exchange, the number of actions needed to transfer information into digital systems or written systems is high for both cases. The same holds for the number of information systems used during the processes. Many of these actions and information systems were introduced to create an overview or to exchange information between parties. Besides the fact that it is time-consuming, these actions induce hazards at different stages of the process. The use of IT to centralise information and to provide information availability for different parties reduces the number of actions and information systems, and hereby, it is expected to reduce the induced hazards. This is also underlined by the fact that more of the high-risk hazards could be controlled by the use of IT support for case I than for case II. The effect of IT support on the number of hazards and on the risk score is not precisely known because no complete HFMEA has been performed on a redesigned supply chain, but we expect that the number, occurrence, and severity of the hazards will decrease. The effect of IT support on hazards related to the delivery of surgical instruments should be assessed in future studies.

In this paper, the processes and hazards were described for only two hospitals and only for orthopaedic surgery. Another limitation is the focus on the supply chain only. Some hazards, mostly in the process step ‘Use in the OR’, are difficult to be controlled by the use of IT, because they are related to cleaning and sterilisation procedures. Examples of such hazards are: ‘An instrument is not cleaned correctly’ and ‘Sterile packaging is damaged’, which are noticed once the instrument tray is opened for use in the OR. A more detailed analysis of the procedures of the CSSD is necessary to be able to identify possible means for IT to control these hazards.

Christian et al. [9] recognised the OR as vulnerable to problems with information exchange leading to delays or extra work for the staff, and recommended to focus on these problems for future patient safety initiatives. The results of the current study are in line with these recommendations, as a large part of the high-risk hazards is expected to be resolved by centralising information and ensuring information availability for different parties. Therefore, it can be inferred that IT support can reduce risks related to unavailability of instruments in the OR. Leape et al. [27] identified process design as a source of medical error, mentioning that many processes in hospitals have not been well thought out as hospitals were never ‘designed’ but just grew. The same is true for the high number of information systems and means of information exchange that was found in this study. Although these systems were introduced to support the exchange of information for the staff, the lack of a structured approach in designing the tools results in increased risks. The supply chain in both hospitals was not designed at once, but is a product of many years of adaptations of the process. When redesigning the supply chain and implementing IT, the necessities of the staff to retrieve information should be taken into account and supported by the IT system. For instance, information about the OR schedule should be centralised and conveniently accessible for all parties, as well as information about the availability of instrument trays, provided by track and trace technology. Moreover, agreements on the tasks and responsibilities of the different parties should be integrated in the redesign of the supply chain.

To conclude, this study revealed a large number of (high risk) hazards in the delivery process of surgical instruments. The majority of the high-risk hazards are expected to be controlled by the use of IT support. Therefore, centralised information and information availability for different parties are expected to reduce risks related to unavailability of instruments in the operating room. The insights gained in this study are a valuable foundation for redesigning the supply chain of surgical instruments.
